# Status of Current Biofluid Biomarkers in Parkinson's Disease

**DOI:** 10.1002/mdc3.13753

**Published:** 2023-05-19

**Authors:** Brit Mollenhauer

**Affiliations:** ^1^ Department of Neurology University Medical Center Göttingen Göttingen Germany; ^2^ Paracelsus‐Elena‐Klinik Kassel Germany

**Keywords:** State, fate, trait, seed amplification assay, α‐synuclein

Biomarkers are crucial to the advancement of therapies for Parkinson's disease (PD). In recent years, significant efforts have been undertaken to identify biomarkers in biological fluids. This has been facilitated through well‐conducted cohort studies that have collected and properly characterized biofluids, as well as through longitudinal follow‐up. More recently, recruitment of prodromal individuals has been initiated. This review provides an update on the current status of biofluid biomarkers in PD.

Biomarkers can serve as indicators of disease state, progression, or predicted clinical course, (ie, markers of state, rate, and fate). All of which would be extremely useful for the management of PD in clinical practice. The high rates of misdiagnosis in clinical practice and in cohort studies, especially in the early disease stages, present a particular challenge. This has been highlighted by a small, but autopsy‐based, study that reported that up to 50% of diagnoses were incorrect within the first 5 years of disease duration.^1^ Diagnostic accuracy based on motor symptoms improves when motor fluctuations, especially dyskinesia, are present; dyskinesia is rarely present in other Parkinson syndromes like multiple system atrophy (MSA) or progressive supranuclear palsy (PSP).^2^

Having a state marker for PD would greatly improve both clinical practice and the clinical trial recruitment of participants in the early disease stages. Similarly, a fate marker that could predict clinical course would not only enhance personalized medicine by improving symptomatic treatment and patient counseling, but would also improve research into different disease phenotypes. Finally, a rate marker is necessary to measure the efficacy of putative neuroprotective agents in clinical trials; a more objective marker than the Unified Parkinson's Disease Rating Scale (UPDRS), for example, is urgently needed to advance therapies.

Biomarker research began earlier in Alzheimer's disease (AD) than in PD, and as such, is more advanced. In AD, an ideal biomarker should reflect a fundamental feature of neuropathology and should be validated in neuropathologically confirmed cases. It should have a diagnostic sensitivity above 80% (for detecting AD) and a specificity of more than 80% for distinguishing other dementias (or Parkinson syndromes in the case of PD). An ideal biomarker should be reliable, reproducible, noninvasive, simple to perform, inexpensive, and investigated by at least two independent studies.^3^ Beyond this definition, the National Institutes of Health have published a list of biomarker endpoints and other tools (BEST) and resources for the categorization of biomarkers with examples of corresponding drug development uses.^4^

In the field of PD, many molecular and biochemical biomarkers have been proposed, but none have been widely accepted or used in routine clinical practice, and none meet the aforementioned criteria of an ideal biomarker. However, the recent emergence of several well‐characterized cohorts and standardized sample collection and processing criteria, along with improved technology (increased sensitivity etc.), has advanced our understanding of PD biomarkers and increased development opportunities.

## Marker of State: Supports the Clinical Diagnosis of PD

The most important non‐fluid biomarkers of state in the diagnosis of PD are dopamine transporter imaging, olfactory testing by smell tests, and rapid eye movement (REM) sleep behavior disorder (RBD) confirmed by video‐polysomnography.

Fluid biomarkers have the potential to reflect molecular disease alterations. There has been considerable effort to detect α‐synuclein (aSyn) in biological fluids, as the aggregation of aSyn is a key element in the pathology of PD. Seed amplification assays (SAA) detecting aggregated aSyn in cerebrospinal fluid (CSF) have recently shown high sensitivity and specificity (~95% for both) for PD in independent cohorts.^5^, ^6^ SAA is a method, detected in the prion field, which amplifies small amounts of protein aggregates in biological fluids when recombinant monomeric protein is added. aSyn SAA is specific for aSyn aggregation disorders including PD, MSA, or dementia with Lewy bodies (DLB). Some reports even find different dynamics in the kinetics of aSyn SAA in CSF in MSA compared to PD.^7^ The signal seems to be specific and could help with difficult differential diagnoses, for example, when there is overlap with tau aggregation disorders such as distinguishing DLB from AD or MSA from PSP.

It has been shown that the positive signal from aSyn SAA appears in those at risk of developing the disease. Positive aSyn SAA signals have been detected in CSF in patients with isolated RBD up to 9 years before conversion to overt PD.^8^ Nevertheless, a lumbar puncture remains an invasive and impractical procedure for clinical routine, especially for screening populations. Therefore, once this technology is available from peripheral sources, and not just from CSF, it will be a valuable tool for screening larger populations and for identifying those at risk of developing PD, especially in upcoming interventional trials. To make this technology more accessible, numerous efforts are underway to establish the aSyn SAA method for biopsies from skin and other tissues, such as the submandibular gland or olfactory mucosa, and other fluids such as blood and saliva: In 2010, Braak and Tredici^9^ described Lewy pathology in the submandibular gland. A recent meta‐analysis revealed that several years ago researchers had already reported decreased total aSyn and increased oligomeric aSyn in the saliva of patients with PD.^10^ More recently, two studies using SAA have also shown positive aSyn SAA in saliva of patients with PD or MSA, raising the hope of developing biomarkers from peripheral sources in the future.^11^, ^12^

## Marker of Rate: Objectively Reflects Disease Progression

A biomarker of rate to objectively reflect disease progression, which is mainly a domain of therapeutic evaluation in routine and clinical trials, has been assessed in recent longitudinal cohort studies. The quantification of total aSyn in CSF using immunoassays has shown a 10% to 15% decrease of levels in PD, as well as in DLB and MSA. However, there is a tremendous overlap of single values in cross‐sectional measures,^13^ as well as consistently across several visits longitudinally (compared to healthy controls). A significant change over time had not been reported. The decrease is not significantly correlated with changes in clinical measures such as motor or cognitive symptoms or dopamine transporter values.^14^

Measurements of neurofilament light chain (NfL) correlate better with clinical progression. This marker of axonal damage was previously quantified (by immunoassay) in CSF. Advanced technology has also improved the sensitivity of the quantification in peripheral blood. Longitudinal plasma levels of NfL in PD patients have shown a slight increase over time, correlating with the progression of motor and cognitive symptoms.^15^, ^16^ Although the effects were still small, it is the first time that this has been seen in a blood marker. These results raise hopes for additional PD biomarkers in blood whereby a combination of three to five individual markers could serve as robust progression biomarkers in the future. A more noticeable increase in NfL levels is seen in those diagnosed with PD at baseline, but who are later found to have a Parkinson syndrome (such as MSA or PSP). NfL levels in the blood are significantly elevated early on and increase significantly over time, making them a useful tool for differential diagnosis.

Given the mounting evidence for early peripheral involvement in PD and the need to introduce neuroprotective therapy much earlier (ie, in individuals at risk) it is crucial to identify biomarkers in peripheral fluids or other fluids or tissues that are more easily collected than CSF, as already mentioned above.

## Marker of Trait: Identifies Individuals at Risk of Developing PD

The identification of individuals at risk of developing PD is of the utmost importance given the failure of previous neuroprotective therapies. This failure is due, in part, to the fact that most neuroprotective therapies are administered at the time of diagnosis when PD is already advanced.

Non‐motor symptoms, such as constipation, RBD, hyposmia, depression/anxiety, and autonomic features, can indicate a high risk of developing PD decades before motor symptom onset. Therefore, efforts are underway to identify individuals at risk, including through cohorts like Parkinson's Progression Markers Initiative online (United States) or Healthy Brain Aging (Europe). The International Parkinson and Movement Disorder Society has generated a web‐based medical calculator for assessing prodromal risk in parkinsonism.^17^ This calculator, which is based on a previous publication,^18^ can be useful for individual risk assessment.

## Outlook

With the availability of longitudinal cohorts that collect biofluid and tissue samples for biomarker research and the advancement of technologies that increase sensitivity and precision, the prospects of identifying new biomarkers have greatly improved. Recent efforts have identified CSF SAA and blood NfL. As these cohorts enable new and innovative biomarker concepts, further biomarkers will follow. Although, CSF SAA is available in some countries for routine testing, most biomarkers still need further validation before they can provide meaningful information to clinicians and patients about the state, rate, and trait of PD.

## Conclusion

The current best diagnostic biomarker for diagnosing aSyn is SAA measured in CSF, whereas the most specific biomarker to determine the risk for developing PD is polysomnography when RBD is suspected. However, more accessible and cost‐effective biomarkers for the prodromal disease stage are needed to establish population‐based cohorts for early detection and intervention.

## Author Roles

(1) Research project: A. Conception, B. Organization, C. Execution; (2) Statistical Analysis: A. Design, B. Execution, C. Review and Critique; (3) Manuscript Preparation: A. Writing of the first draft, B. Review and Critique.

B.M.: 3A and 3B.

## Disclosures

**Ethical Compliance Statement:** Informed patient consent was not necessary for this work, nor was the approval of an institutional review board. We confirm that we have read the Journal's position on issues involved in ethical publication and affirm that this work is consistent with those guidelines.

**Funding Sources and Conflicts of Interest:** None related to this article.

**Financial Disclosures for Previous 12 Months:** In the last 12 months, B.M. has received honoraria for consultancy from Roche, AbbVie, Biogen, and Sun Pharma Advanced Research Company. B.M. is member of the Executive Scientific Advisory Board of The Michael J. Fox. Foundation for Parkinson's Research (MJFF) and of the steering committee of the Parkinson Progression Marker Initiative and co‐PI of the Systemic Synuclein Sampling Study and member of the Therapeutic Evaluation Committee (TEC) of the Path to Prevention of MJFF. She has received grants from the Deutsche Forschungsgemeinschaft (DFG), BMBF, EU (Horizon2020), Parkinson Fonds Deutschland, and Deutsche Parkinson Vereinigung, MJFF.
